# Trauma-Informed Care in Digital Health Technologies: Protocol for a Scoping Review

**DOI:** 10.2196/46842

**Published:** 2023-06-23

**Authors:** Abdul-Fatawu Abdulai, Hasti Naghdali, Eden Tekie Ghirmay, Fuseini Adam, Eunice Bawafaa

**Affiliations:** 1 School of Nursing, Faculty of Applied Sciences, University of British Columbia Vancouver, BC Canada; 2 Integrated Sciences, University of British Columbia Vancouver, BC Canada; 3 Lawrence S Bloomberg Faculty of Nursing, University of Toronto Toronto, ON Canada

**Keywords:** clinical intervention, digital health technologies, digital health, psychological trauma, stress, trauma, trauma-informed care

## Abstract

**Background:**

The use of digital health technologies is becoming increasingly common across the globe as they offer immense potential to enhance health care delivery by promoting accessibility, flexibility, and personalized care, connecting patients to health care professionals, and offering more efficient services and treatments to remote residents. At the same time, there is an increasing recognition of how digital health can inadvertently foment psychological trauma. This phenomenon has led to the adoption of trauma-informed care in designing and deploying digital health technologies. However, how trauma-informed care is defined and characterized, and the various trauma-informed care strategies used in designing and deploying digital health technologies remain unexplored.

**Objective:**

This scoping review aims to explore and synthesize the literature on how trauma-informed care is defined and characterized in digital health and the various trauma-informed care principles, strategies, or recommendations used in designing and deploying digital health.

**Methods:**

This review will draw on the Joanna Briggs Institute’s updated methodological guidance for scoping reviews. A search will be conducted on CINAHL, PubMed, Embase, Compendex Engineering Village, Web of Science, Scopus, and PsycINFO. This review will consider published research studies and unpublished work (gray literature). Studies will be included if they applied trauma-informed care in designing or deploying digital health for patients across all geographical locations or provide trauma-informed recommendations on how web developers should develop digital health. Studies will be limited to publications within the past 10 years and studies in all languages will be considered. Two independent reviewers will screen the titles and abstracts, and then perform a full-text review. Data will be extracted into a data extraction tool developed for this study.

**Results:**

The scoping review was undergoing a full search as of April 2023. The main results will synthesize the peer-reviewed and gray literature on adopting trauma-informed care practices in digital health research and development. The study is expected to be completed by December 2023 and the results are expected to be published in a peer-reviewed journal.

**Conclusions:**

This review is expected to provide the knowledge base on the adoption of trauma-informed care in designing and deploying digital health. This knowledge can lead to more engaging, and likely, more effective digital health interventions that have less potential for harm. A synthesis of the various trauma-informed care strategies in digital health will also provide a trauma-informed language by enabling researchers and digital health developers to consider trauma as a critical factor in each stage of the design process.

**International Registered Report Identifier (IRRID):**

DERR1-10.2196/46842

## Introduction

### Overview

Trauma is a term used to describe the challenging emotional and psychological consequences associated with an individual after going through a distressful event [1]. This definition of trauma is specific to emotional and psychological trauma, which is different from physical trauma (ie, injuries or body wounds produced by sudden physical injury from impact, violence, or accident). According to the World Health Organization, approximately 70% of people across the globe have experienced at least one traumatic event with varying emotional and psychological sequelae [2]. The number of reported cases of trauma increased across several countries during the COVID-19 pandemic [3]. Living through the COVID-19 pandemic has heightened the degrees of trauma with symptoms that can eventually harm a person’s sense of safety and distort their ability to regulate emotions and navigate relationships [4]. At the same time, the outbreak of the pandemic saw an accelerated adoption of digital health for various clinical and public health services across the globe. In the United States, telemedicine appointments through “Plush Care” and “American Well” increased by 70% and 150% respectively [5]. In Canada, the number of people using digital health interventions increased from a prepandemic level of 72% to approximately 86% in some provinces during the peak of the pandemic [6,7]. Technologies such as Skype, WhatsApp, and FaceTime were adapted as alternative and complementary methods of health care delivery and health care access across Australia and various European countries [8,9]. While digital health is convenient and cost-effective, there are legitimate concerns about how the digital transformation of health care can lead to unintended consequences, including triggering or perpetuating trauma among end users [10]. The likelihood of perpetuating unintended consequences like trauma through digital technologies may be higher in patient-facing digital health interventions than in clinician-facing technologies. This is possible because, unlike technologies in clinical settings, patient-facing technologies (eg, patient portals, mHealth, and health-related websites) are more likely to be unregulated and unmoderated. Bonell et al [11] termed this the “dark logic” of interventions where digital health could result in unintended trauma-related consequences despite a generally virtuous intention.

### Technology-Mediated Trauma

Trauma resulting from the use of digital health technologies (herein known as technology-mediated trauma) is becoming common with the increasing use of digital health interventions [12-14]. While technology-mediated trauma started emerging in social media platforms [15-17], emerging evidence shows that digital health technologies, especially patient-facing digital health technologies can also trigger or facilitate technology-mediated trauma depending on its’ interface design [18,19]. Indeed, technology-mediated trauma perpetrated through digital health platforms may produce worse outcomes due to the potentially vulnerable nature of people who uses such technologies (ie, patients).

Technology-mediated trauma can occur in various forms. For instance, interventions delivered through web platforms that unexpectedly expose people’s information or identities can cause reputational and psychological damage with associated trauma-related symptoms. Also, digital health technologies (eg, HIV/AIDS web-based platforms and social networking sites for people with mental health problems) that expose people to sextortion, identity theft, cyberbullying, and web-based scams, including the purchase of fake health products, could result in short-term or long-term emotional traumatic consequences, and sometimes, suicidal thoughts [20,21]. Other forms of digital communication channels embedded within digital health platforms, such as chatrooms, user subscription channels, forums and bulletin boards, social networking sites, and other platforms that enable information sharing, may also be used unscrupulously to victimize, and expose users of digital platforms in a manner that could result in technology-mediated trauma-related experiences [22-26]. While youth with mental health challenges found the use of social networking sites to be highly usable, engaging, and supportive [27], such sites could also pose avenues for web-based victimization and bullying with the potential consequences for trauma-related experiences [28]. For instance, cyberbullying activities perpetrated through web-based social networking sites for mental health patients, including negative forum posts and negative comments, have been shown to result in technology-mediated trauma experiences and other forms of mental health challenges among adults [28,29]. Web-based practices such as advertisements carried out on digital platforms like sexual health–related websites can inadvertently foment stigma [30]. For instance, discriminatory advertising on maternal health websites can inadvertently perpetuate trauma when a woman who just experienced a miscarriage is not able to opt out of pregnancy adverts when using such a platform [31]. Technology-mediated trauma experiences can result in devastating sequelae for people’s overall psychological and physical well-being [32]. The negative effects of technology-mediated trauma on psychological well-being were demonstrated in a recent systematic review that revealed a negative association between digital technology use and emotional well-being outcomes among teenagers during the COVID-19 pandemic [33].

The pervasive and potentially negative impact of technology-mediated trauma on end users made it necessary within the digital health community to rethink “the usual way of doing business” by incorporating trauma-informed care in the design and deployment of digital health interventions. Some researchers subsequently coined the term “trauma-informed design” to denote the application of trauma-informed care in developing interactive technologies, including digital health platforms.

### Trauma-Informed Care in Digital Health

Trauma-informed care in interactive digital technologies is a new concept that started appearing as blog posts among user interface and user experience practitioners across the United States, the United Kingdom, and Australia. The concept of trauma-informed design became prominent in the later parts of 2020 [34]. Since then, researchers have been actively engaged in developing approaches in which trauma-informed principles, approaches, or practices can be translated into the design of digital health technologies to make them emotionally safer. Trauma-informed care recognizes the effects of trauma on users of digital health technologies and provides theoretical approaches and practical guidance for how such technologies can be designed to be emotionally safe and empowering for all users [34]. Indeed, several digital health interventions have adopted trauma-informed approaches in designing and developing digital health interventions or produced trauma-informed recommendations that can be used to adapt old interventions or develop new digital health services [35-38]. Other researchers have developed trauma-informed web heuristics for designing human-centered information systems, including digital health [39].

While trauma-informed care is gradually gaining ground in the digital health community, how the concept is defined and characterized, and the various trauma-informed care strategies used in designing and deploying digital health technologies remain unexplored and ill-understood. The purpose of this review is two-fold: (1) to explore and synthesize the current state of the literature that has defined and characterized trauma-informed care in digital health and (2) to synthesize the literature on various trauma-informed care strategies, practices, principles, or approaches that are used in designing and deploying digital health. A preliminary search was conducted in February 2023 in Cochrane Databases, the Joanna Briggs Institute (JBI) Evidence Synthesis, and PROSPERO to identify any possible reviews that might have been conducted on the topic. The search results did not reveal any scoping or systematic review that has been conducted on our proposed topic. However, the search did return relevant articles that were used in formulating our search terms [13,35-38].

### Review Questions

1. How is trauma-informed care defined and characterized in the digital health literature?
2. What trauma-informed care principles, strategies, or recommendations are currently used in designing and deploying digital health technologies?

## Methods

### Overview

This proposed review will draw on the JBI updated methodological guidance for the conduct of scoping reviews [40]. We will use the PRISMA-ScR (Preferred Reporting Items for Systematic Reviews and Meta-analyses Extension for Scoping Reviews) framework to report the findings of this review [41]. Because trauma-informed care in digital health is an emerging area with varied sources of evidence, we consider a scoping review to be more appropriate in providing conceptual clarity on the topic than a systematic review.

### Inclusion and Exclusion Criteria

The inclusion and exclusion criteria for this proposed review will adopt the JBI criteria of population, concepts, and context outlined by Peters et al [40]. The details of each criterion are outlined below.

### Population

This review will include 2 distinct populations. These include (1) digital health technology designers (eg, software developers, content creators, and user interface and user experience professionals) and (2) patients who use digital health technologies (without consideration to a type of patient or disease or age). For the first population, studies will be included if they provide trauma-informed strategies or recommendations for developers or designers of digital health technologies. For the second population, studies will be included if they explore ways in which trauma-informed care principles or strategies can be applied in designing or deploying digital health technologies for patients. Studies will also be included if they explore how trauma-informed care can be applied to digital technologies to improve patients’ health outcomes. A patient in this study is used to refer to anyone who uses a digital health intervention for health-related purposes. Digital health technology studies on posttraumatic stress disorder (PTSD) that adopt trauma-informed care approaches or provide trauma-informed care recommendations will be included. However, digital health technology studies on PTSD that did not adopt or recommend trauma-informed care approaches will be excluded.

### Concepts

This review aims to map the evidence on how trauma-informed care is defined and characterized in digital health and to synthesize the literature on trauma-informed care in designing and deploying digital health. Therefore, the review will include two major concepts: (1) trauma-informed care and (2) digital health technologies. Based on these concepts, we will consider studies that discussed the definitions and characteristics of trauma-informed care in digital health. We will also consider studies that provided trauma-informed care strategies, practices, approaches, or recommendations for designing and deploying digital health. Studies that provide trauma-informed care strategies which are not focused on designing or deploying digital health technologies will be excluded. In this review, trauma is limited to psychological trauma at the personal and interpersonal levels. Other forms of trauma, such as physical trauma, group trauma, community trauma, or mass trauma or casualties, are beyond the scope of this review. Digital health in this review is used to refer to patient-facing technologies, including mHealth, wearable devices, health-related websites, patient portals, social networking sites, etc. Digital health technologies that are focused on health care professionals (eg, electronic health records and clinical decision support systems) will be excluded from this review. We decided to exclude clinician-facing technologies because such technologies are more likely to be regulated and might have fewer chances of causing trauma among patients compared to patient-facing technologies.

### Context

This review will include studies that are conducted with individuals in all age groups in any geographical location. Our search will not be limited to any specific gender or sex group, a particular disease condition, and a specific user group or population. We aim to include all outpatient or nonclinical settings that explore how trauma-informed care can be applied in designing or deploying digital health technologies (community health centers, social service organizations, etc), and industry (technology design companies). We will also consider digital technology developers (eg, User Experience research centers) that have discussed, developed, or applied trauma-informed care principles in designing digital health.

### Types of Evidence

This scoping review will include all types of peer-reviewed studies on trauma-informed care in digital health, including primary research studies (quantitative, qualitative, and mixed methods research) and review papers (systematic reviews or meta-analyses). We will also consider gray literature on trauma-informed care in digital health, including theses, editorials, commentaries, government reports, industry-sponsored works, guidelines, book chapters, and blogs. Abstracts, letters to editors, and short papers will be excluded. Gray literature is considered important because trauma-informed care strategies or recommendations for designing digital health may be developed and published outside traditional academic publishing and distribution channels. These include industry-sponsored works that may provide trauma-informed recommendations (software designers, software engineers, etc) but may not necessarily be published in peer-reviewed journals. Due to the emerging nature of the field and the rapid advancements in digital health technologies in the past decade [42], this review will be limited to studies conducted within the past 10 years.

### Search Strategy

A strategy was developed in consultation with a university librarian at the University of British Columbia. Following Pollock’s [43] practical guide to conducting scoping reviews, a preliminary search was conducted in Cochrane Databases, the JBI Evidence Synthesis, and PROSPERO to determine if any reviews were conducted on the topic and to identify additional words and phrases to inform the search strategy. Given the multidisciplinary nature of the topic (ie, trauma-informed care and digital health), we formulated a search strategy that reflected each dimension of the topic. The search strategy for the gray literature will be iterative depending on the emerging evidence. Using the refined search strategy, a literature search will be conducted on a variety of databases, including CINAHL, PubMed, Embase, Compendex Engineering Village, Web of Science, Scopus, and PsycINFO. These databases were selected because they covered a broad range of published literature that reflected the topic in nursing, medicine, psychology, allied sciences, engineering, and computer science. A reference list of articles that meet the inclusion criteria will be manually screened to identify additional eligible studies that were not returned by the search strategy. Using different search terms, we will also search for gray literature through Google and Google Scholar. We will open individual web links that contain blogs or industry materials on trauma-informed care in digital health. Multimedia Appendix 1 contains the detailed search strategy as well as the data extraction guide for both published and grey literature.

A preliminary search conducted on the 2 databases for gray literature produced results of less than 5 pages each. Since we do not expect these databases to produce results that exceed 10 pages by the time the review is completed (ie, December 2023), we are limiting the data from Google and Google Scholar to the first 100 results. Consistent with JBI recommendations, we will not apply language restrictions to our search. We will use translation services for eligible studies that fall outside the proficiency of the authors. Where necessary, the authors of relevant publications, blogs, and opinion pieces will be contacted to get additional information or seek clarification regarding their publications. As an emerging area of research, it is possible that some latest evidence might have been published after our literature search. To identify any latest publication, we will repeat the search strategy (but limited to the specific time frame) before the final manuscript is submitted for publication.

### Study Selection

Search results from all databases will be exported into Endnote X9 (Clarivate, 2020). Search results from Google and Google Scholar will be manually added and duplicates will be removed accordingly. Two independent reviewers will then screen the titles and abstracts using the inclusion and exclusion criteria outlined under the population, concepts, and contexts. Studies that meet the inclusion criteria will undergo a full-text review by the 2 independent reviewers. Any discrepancies that result from either the title, abstract, or full-text review between the 2 reviewers will be solved through a discussion with a third reviewer.

### Data Extraction

Specific information from the included studies will be extracted by the 2 independent reviewers. The data will be summarized on an Excel spreadsheet developed in line with the JBI guidelines for data extraction [43]. The data extraction sheet will contain specific information about each study, including the authors’ names, the study’s title, the year of publication, geographical location, the study population, type of digital health technology, target population, sample size (if applicable), purpose, and the findings of each study. Where applicable, information from each paper will be entered into the respective portion of the spreadsheet. We will also extract information specific to each of the 2 populations (ie, health technology developers and patients). For studies related to how technology developers adopt trauma-informed care, we will extract data related to the specialty, and place of work or origin (eg, industry and research). For studies on patient populations, we will extract data on age groups, sex and gender, socioeconomic status, and health issues. The data extraction tool will be amended for gray literature and will be iterative depending on the kind of emerging evidence. To reduce biases and ensure consistency and comprehensiveness of the data extracted, the 2 reviewers will each conduct independent data extraction on the first 4 papers (both published and gray literature). These preliminary data will be compared, contrasted, and discussed with a third reviewer to resolve any disagreements before they are applied to the rest of the papers. The remaining data extraction will occur through an ongoing team discussion and disagreements will be resolved by discussion with a third reviewer.

### Data Analysis and Presentation

The search results as well as the inclusion and exclusion pathways for various studies will be reported in line with the PRISMA-ScR flow diagram [44]. The extracted data and the themes will be presented as narrative summaries. This will be augmented by numerical summaries to report each high-level domain that is common across the papers. Such numerical summaries may include the number of papers that adopt trauma-informed approaches and the number that provide trauma-informed recommendations. It may also include a summary of the number of trauma-informed guidelines and their area of focus in the digital health lifecycle (ie, the needs assessments, design, evaluation, or deployment phases of digital health). Research and gray literature will be grouped accordingly and papers that reflected the 2 research questions will also be grouped in the numerical summaries. To answer the research questions, the included papers will be analyzed thematically using inductive coding by 2 reviewers. Depending on the number of papers that meet the inclusion criteria, 3 papers will be reviewed and coded independently by 2 reviewers for each research question. The different coding frameworks will then be discussed among the reviewers to arrive at a final coding structure for each research question. The coding structure will then be applied to the rest of the papers to identify emerging themes. The emerging themes will then be clustered into major themes that represent the definitions of trauma-informed care in digital health and trauma-informed care strategies for designing and deploying digital health technologies.

## Results

The scoping review was undergoing a comprehensive literature search at the time this protocol was being published. This will be followed by tile and abstract screening and then full data extraction. The scoping review is expected to be completed and the manuscript submitted to a peer-review journal by December 2023.

## Discussion

### Overview

Trauma-informed care in digital health is an emerging area of research that sought to explore various trauma-informed approaches in developing and deploying digital health interventions that limit the potential for further harm to end-user patients. There are several systematic and scoping reviews on trauma-informed care in various aspects of health care as well as in the built environment [37,45]. Despite an increasing application of trauma-informed care principles in digital health research, no reviews summarizing the definitions or characterization of trauma-informed care approaches in digital health were found. This scoping review was conceptualized in October 2022 to summarize the current state of the literature on trauma-informed care in digital health. This scoping review will form an evidence base for an emerging program of research on trauma-informed design in digital health. The results of this scoping review are expected to provide researchers with an extant knowledge of how trauma-informed care is defined, characterized, and adopted in digital health while providing directions for future research on the topic. Digital health technologies are increasingly developed for managing various traumatic stress and PTSD [46,47]. Therefore, we believe that this review will provide trauma-informed recommendations for designing health technologies that have the least chance of causing harm while ensuring emotional safety to end users.

### Limitations

Abstracts, letters to editors, and short papers that might have been relevant to the topic will be excluded. Secondly, the methodological quality of this review will not be assessed. Therefore, we cannot conclude whether the trauma-informed strategies that would be synthesized from this review are indeed effective or applicable to digital health. While we acknowledged these limitations, we believe that they would not diminish the quality of our review because scoping reviews by their nature do not assess the methodological quality of studies.

### Conclusions

This review is expected to provide the knowledge base on the definition, characterization, and adoption of trauma-informed care in designing and deploying digital health interventions. This knowledge can lead to more engaging, and likely, more effective digital health interventions by enabling researchers and digital health developers to consider trauma as a critical factor in each stage of the design and deployment process. A synthesis of the various trauma-informed care strategies used in digital health will also provide a trauma-informed language for developing digital health technologies that have less potential for harm.

## Appendix

Multimedia Appendix 1 Search terms and data extraction.

## Abbreviations

JBI: Joanna Briggs Institute
PRISMA-ScR: Preferred Reporting Items for Systematic Reviews and Meta-analyses extension for scoping review
PTSD: posttraumatic stress disorder
