# Supplementary material for: Trauma-Informed Care in Digital Health Technologies: Protocol for a Scoping Review
Source: JMIR Res Protoc. 2023 Jun 23;12:e46842. doi: 10.2196/46842 (PMC10337410; doi:10.2196/46842)
Supplement: Multimedia Appendix 1 [file resprot_v12i1e46842_app1.docx]

**Multimedia Appendix 1**

**Table 1: S1. Preliminary Data Base Search Strategies**

Note: Search terms are slightly modified for each database

| ***Database*** | ***Search Terms*** |
| --- | --- |
| *CINAHL*  Scopus | *Digital health” OR “Digital Technology” OR “eHealth” OR “telehealth’ OR “telemedicine” OR “mHealth” OR “uhealth” “Internet” OR “website” OR “web application” OR “internet searching” OR “web development” OR “worldwide web*  ***AND***  *“Trauma-informed design” OR “Trauma-informed care” OR “trauma-informed practices” OR trauma-informed approach” OR “trauma-informed approaches” OR “post-traumatic stress” OR “PTSD” OR “interpersonal trauma” OR “traumatic violence”* |
| *MEDLINE (Embase),* PubMed,  Web of Science | *(internet.mp. or Internet/ or internet access/ OR Internet/ or medical information/ OR Worldwide web.mp. or Internet/ OR eHealth.mp. or telehealth/ OR mobile application/ or telemedicine/ or mobile phone/ or mhealth.mp. OR telemedicine/ or telenursing/ or teleconsultation/*  ***AND***  *“Trauma” OR “Post-Traumatic Stress Disorder” OR “Traumatic stress” OR “Trauma-informed care’ OR “Trauma-Informed approaches” OR “Trauma and violence informed care”* |
| *Engineering Village* | *“Internet OR website OR web application OR internet searching OR web development OR worldwide web OR eHealth OR mHealth OR telehealth OR telenursing OR ubiquitous health*  ***AND***  *Trauma-informed design” OR “Trauma-informed care” OR “trauma-informed practices” OR trauma-informed approach” OR “trauma-informed approaches” OR “post-traumatic stress” OR “PTSD” OR “interpersonal trauma” OR “traumatic violence.* |
| *Psych INFO* | *mHealth, OR wearable devices OR health-related websites OR patient portals, social networking sites OR web application OR internet searching OR web development OR worldwide web OR eHealth OR mHealth OR telehealth OR telenursing OR ubiquitous health*  ***AND***  *Trauma-informed design” OR “Trauma-informed care” OR “trauma-informed practices” OR trauma-informed approach” OR “trauma-informed approaches” OR “post-traumatic stress” OR “PTSD” OR “interpersonal trauma” OR “traumatic violence* |

**Table 2: S2. Data Extraction Tool for Peer Review Publications**

| **Domain** | **Description** |
| --- | --- |
| Title & Name of Authors | Indicate the name of the authors |
| Year of Publication | Indicate the year of publication |
| Title of Publication | Indicate the full title of the article |
| Type of publication | Identifies the article if it is an original research study, review paper, or grey literature |
| Source | Identifies the name of the journal/ blog/ commentary? |
| Aims/Objectives | State the aims and objectives as indicated in the paper |
| Geographical location | Indicate the country where the study was conducted or the grey literature was written. |
| Sample characteristics | Indicate the group of health workers involved in the study |
| Sample size | Indicate the number of participants in the study |
| Study design | Indicate the methodology adopted e.g. qualitative, quantitative, mixed-method, case study, review. |
| Type of digital health | Indicate the type of digital health technology/intervention |
| Target population | Indicate if the focus of the study is on developers of digital health interventions or the focus is on patients. |
| If the population include patients | Indicate age groups, sex and gender, socioeconomic status, and health issues |
| Key findings | Describe the main results (evidence, concept, themes) and link to the scoping review question and objectives |
| Key themes related to the definition of trauma-informed care in digital health | Indicate the definition of trauma-informed care if identified in any part of the paper. |
| Key themes related trauma-informed care principles/strategies in digital health digital health | Identify the domains of trauma-informed care principles, strategies, or recommendations as used in digital health |
| limitations | Indicate the limitations of the paper |

**Table 3 S3 Sample Data Extraction Tool for Grey Literature**

| **Domain** | **Description** |
| --- | --- |
| Title & Name of Authors | Indicate the name of the authors |
| Year of Publication | Indicate the year of publication |
| Title of Publication | Indicate the full title of the publication |
| Type of publication | Identifies the source (letters to editors, commentaries, government reports, guidelines, book chapters or a blog) |
| Source of origin | Identifies the source name (is it from the industry, academic, healthcare organization, government, or independent investigators) |
| Aims/Objectives | State the aims and objectives as indicated in the paper |
| Geographical location | Indicate the country where the work was conducted and/or published. |
| Sample characteristics | Indicate the group of health workers involved in the study |
| Sample size | Indicate the number of participants in the study |
| Study design | Indicate the methodology adopted e.g. qualitative, quantitative, mixed-method, case study, review. |
| Type of digital health | Indicate the type of digital health technology/intervention |
| Target population | Indicate if the population is digital health developers and/or patients |
| If the population include patients | Indicate age groups, sex and gender, socioeconomic status, and health issues |
| Key findings | Describe the main results (evidence, concept, themes) and link to the scoping review question and objectives. |
| Key themes related to the definition of trauma-informed care in digital health | Indicate the definition of trauma-informed care if identified in any part of the paper. |
| Key themes related trauma-informed care principles/strategies in digital health digital health | Identify the domains of trauma-informed care principles, strategies, or recommendations as used in digital health |
